# Coexpression Network Analysis of Benign and Malignant Phenotypes of SIV-Infected Sooty Mangabey and Rhesus Macaque

**DOI:** 10.1371/journal.pone.0156170

**Published:** 2016-06-09

**Authors:** Zhao-Wan Yang, Yan-Hua Jiang, Chuang Ma, Guido Silvestri, Steven E. Bosinger, Bai-Lian Li, Ambrose Jong, Yan-Hong Zhou, Sheng-He Huang

**Affiliations:** 1 Hubei Bioinformatics and Molecular Imaging Key Laboratory, College of Life Science and Technology, Huazhong University of Science and Technology, Wuhan, China; 2 Saban Research Institute of Children’s Hospital Los Angeles, Department of Pediatrics, University of Southern California, Los Angeles, California, United States of America; 3 Yerkes National Primate Research Center, Department of Pathology and Laboratory Medicine, Emory University School of Medicine, Atlanta, Georgia, United States of America; 4 Ecological Complexity and Modeling Laboratory, University of California Riverside, Riverside, CA 92521–0124, United States of America; University of Pittsburgh Center for Vaccine Research, UNITED STATES

## Abstract

To explore the differences between the extreme SIV infection phenotypes, nonprogression (BEN: benign) to AIDS in sooty mangabeys (SMs) and progression to AIDS (MAL: malignant) in rhesus macaques (RMs), we performed an integrated dual positive-negative connectivity (DPNC) analysis of gene coexpression networks (GCN) based on publicly available big data sets in the GEO database of NCBI. The microarray-based gene expression data sets were generated, respectively, from the peripheral blood of SMs and RMs at several time points of SIV infection. Significant differences of GCN changes in DPNC values were observed in SIV-infected SMs and RMs. There are three groups of enriched genes or pathways (EGPs) that are associated with three SIV infection phenotypes (BEN^+^, MAL^+^ and mixed BEN^+^/MAL^+^). The MAL^+^ phenotype in SIV-infected RMs is specifically associated with eight EGPs, including the protein ubiquitin proteasome system, p53, granzyme A, gramzyme B, polo-like kinase, Glucocorticoid receptor, oxidative phosyphorylation and mitochondrial signaling. Mitochondrial (endosymbiotic) dysfunction is solely present in RMs. Specific BEN+ pattern changes in four EGPs are identified in SIV-infected SMs, including the pathways contributing to interferon signaling, BRCA1/DNA damage response, PKR/INF induction and LGALS8. There are three enriched pathways (PRR-activated IRF signaling, RIG1-like receptor and PRR pathway) contributing to the mixed (BEN+/MAL+) phenotypes of SIV infections in RMs and SMs, suggesting that these pathways play a dual role in the host defense against viral infections. Further analysis of Hub genes in these GCNs revealed that the genes LGALS8 and IL-17RA, which positively regulate the barrier function of the gut mucosa and the immune homeostasis with the gut microbiota (exosymbiosis), were significantly differentially expressed in RMs and SMs. Our data suggest that there exists an exo- (dysbiosis of the gut microbiota) and endo- (mitochondrial dysfunction) symbiotic imbalance (EESI) in HIV/SIV infections. Dissecting the mechanisms of the exo-endo symbiotic balance (EESB) that maintains immune homeostasis and the EESI problems in HIV/SIV infections may lead to a better understanding of the pathogenesis of AIDS and the development of novel interventions for the rational control of this disease.

## Introduction

Progressive AIDS caused by the human immunodeficiency virus type 1 (HIV) and simian immunodeficiency virus (SIV) is characterized by systemic inflammation, opportunistic infection and malignant disorders resulting from generalized immune activation-mediated destruction of the host defense system [1–3]. Although tremendous progress has been made in the fighting against AIDS since the discovery of this disease 1981, there is currently no effective vaccine or cure available for AIDS today [4–5]. Pathogenesis, prevention, treatment and cure of HIV-1 infection remain one of the greatest challenges in modern medicine due to its ability to mutate very quickly, and to hide within cells from both drugs and the immune system, which leads to persistent viral infection/immune activation and microbial translocation, and eventually progresses to AIDS. Understanding of the differences in phenotypes of HIV infection may be very important for uncovering this relationship and conquering AIDS. There are usually two basic phenotypes [malignant (MAL) and benign (BEN)] of microbial infection including human HIV/AIDS [6]. The two extreme phenotypes of HIV and SIV infection include slow or rapid progression to AIDS (MAL) in a majority of the infected human population and the non-natural primate host (i.e., rhesus macaques, RMs), and nonprogression to AIDS (BEN) in a minority of the infected human population and the natural primate hosts (i.e., sooty mangabeys, SMs) [7]. Studies on SIV infection in nonhuman primates (NHP) have offered promise and advantages for gaining new insights into the pathogenesis of HIV/AIDS [7–14].

The nature of host-microbe relationships is critical for development of microbial infections including AIDS [15–18]. Microbial infection is an ecological and evolutionary paradigm, which is associated with co-evolution between hosts and microbes in dynamic ecosystems [6,15,18]. Nonpathogenic microbiota, the major microbial community, forms a healthy symbiotic ‘superorganism’ with the hosts [16]. There are two types of symbiosis (Sym), exosymbiosis (e.g, microbiota) and endosymbiosis (e.g., mitochondria). It has been suggested that the exo-endo Sym balance (EESB) highly contribute to maintain the host homeostasis [18]. From birth to death, a symbiotic relationship has been established and maintained between the host and a vast, complex, and dynamic consortium of microbes [16–18]. Most of our microbial commensals with up to 100 trillion (10^14^) microbes reside in the gastrointestinal (GI), which is the largest mucosal lymphoid organ in the body with a very large percentage of the immune cells [18–19]. GI abnormalities, such as diarrhea, weight loss, and malnutrition, have been shown to occur in HIV-1 and SIV-infected individuals. In the early phase of HIV/SIV infection, disruption of the intestinal epithelial barrier is characterized by apoptosis, changes in gene expression associated with epithelial barrier functions as well as upregulation of inflammatory genes [19–20]. The GI integrity is disturbed with concurrent disruption of the mucosal immune system that is characterized by a significant and substantial loss of mucosal CD4 T cells. This process persists throughout the course of viral infection. The major consequence of the disturbed GI integrity is the increased translocation of microbes and their products that would normally be present within the intestinal lumen into the lamina propria, draining lymph nodes, and ultimately the systemic circulation. Disturbed exosymbiosis (dysbiosis) has been observed in HIV- and SIV-infected individuals with a disproportionate amount of Proteobacteria within the microbiome that is a common hallmark in diseases with the involvement of inflammation within the GI tract. In conjunction with this microbial translocation, HIV/SIV infection is associated with increased immune activation [19–21]. The disturbed mucosal community of microorganisms is shown to be correlated with a number of markers of disease progression, systemic inflammation, and upregulation of the tryptophan catabolism pathway, which is a crossroad between microbes and host [22]. During the course of HIV infection, a significant and substantial depletion of mucosal CD4 T cells occurs in conjunction with significant declines in T helper 17 (Th17) cells. These T cells contribute to intestinal barrier homeostasis as well as to mucosal defense.

Based on the two extreme phenotypes (BEN and MAL) of HIV and SIV infections [7], we have proposed that HIV/SIV infection is a two-way paradigm (BEN and MAL), not a one-way paradigm (MAL), the conventional wisdom in medicine holding that microbial infection is a pathogenic process [17–18]. The emphasis is on the antagonism or conflict, not the symbiotic relationship. It underrates the effects of exosymbiotic (e.g., microbiota)/endosymbiotic (e.g., mitochondria) factors on microbial infection and hinders the ability to develop rational interventions to cure AIDS [18,23]. Mitochondrial disorders have been found to contribute to the pathogenesis and therapeutics of HIV infection [21]. Mitochondria can directly influence the progression of AIDS, including the viral infectivity, the course of HIV-1 infection, and the prevalence of side effects from the primary HIV-1 therapy, highly active antiretroviral therapy (HAART) since this organelle play a key role in the production of energy and the induction of cellular apoptosis [21]. The common features of gene coexpression networks during HIV infection are the significant changes in the genes with negative connectivity. Currently, the progression of HIV/AIDS, the mechanistic connection between the BEN and MAL phenotypes, and the imbalance between exosymbiosis and endosymbiosis are unknown. Therefore, it remains to be elusive whether or not there is a mechanistic connection between exosymbiosis and endosymbiosis.

The SIV infection of non-natural hosts typically progresses to the AIDS similar to the HIV infection. In contrast, the SIV infection of natural hosts is typically lack of this progression, although it also displays high-level virus loads. Comparative studies between the SIV infection of natural and non-natural hosts can help us to get novel insights into the pathogenesis and therapeutics of HIV/AIDS [24–27]. The transcriptome-wide gene expression provided by microarray technologies provides a valuable resource to perform this comparative analysis [28]. Differential expression analysis has revealed several differences between the SIV infection of natural and non-natural hosts at the gene level [7,11–12]. However, this method ignores the strong coexpression relationships between different genes, and leads to the molecular mechanisms of SIV/HIV infection underlying in gene expression data only partially exploited. Gene coexpression network (GCN) analysis can produce a network to elucidate coexpression patterns of hundreds of or thousands of genes at the system level, thus provides an alternative and powerful approach for further investigating this disease. In the GCN, each node represents a gene, and the line indicates the coexpression relationship between two genes. Genes with high connectivity (i.e., Hub genes) indicate that they may be biologically important in the analyzed disease stage because highly connected hub nodes are positioned at the centers of the network’s architecture [28]. The effectiveness of such gene coexpression network analysis has been demonstrated in the application of understanding molecular mechanisms of various diseases, including HIV infection [23], Alzheimer’s disease [29], obesity [30], schizophrenia [31], etc.

The goal of this study is to find hub genes whose expression profiles correlate positively with the extreme phenotypes of SIV infections. We employed the GCN analysis on the microarray-based gene expression data from sooty mangabeys (SMs) and rhesus macaques (RMs) infected with the same SIVsmm virus strain. As the natural host of SIVsmm, SMs are found to be extensively different from RMs (i.e., non-natural hosts of SIVsmm) in several aspects at the system level. Firstly, the changes of positive and negative connectivity in SM-specific GCNs greatly differ from those in RM-specific GCNs during SIV infection. Secondly, the distribution of genes in the GCNs among three distinct groups varies during the infection and presents a maximum contrary between SM- and RM-specific GCNs at 14 days after SIV infection. Thirdly, selected Hub+ genes usually outnumbers Hub- genes in SM-specific GCNs (See definitions of Hub+ and Hub- genes in Materials and Methods), which is contrary to RM-specific GCNs. Fourthly, interesting differences are observed on genes with the highest connectivity in GCNs, which might be important candidate genes for further investigating pathogenesis of SIV infection. Integrating with resources such as Ingenuity Pathway Analysis system, our further analysis of Hub+/Hub- genes in these GCNs reveals that SIV-infected SMs and RMs exhibit substantial differentials on patterns of enriched pathways, correlation between gene expression and CD4+ T cell level, as well as immune gene and transcription factor number in Hub genes.

## Results

### Data preprocessing and Analysis Design

After data preprocessing, individual probe sets of 428 (SMs) and 941 (RMs) were identified as having significantly differential expressions based on the p-value (<0.05) of one-way ANOVA test (Benjamini-Hochberg FDR multiple test correction) and fold-change value of gene expression.

### Global differences in SM- and RM-specific GCNs during SIV Infection

**Fig 1:**
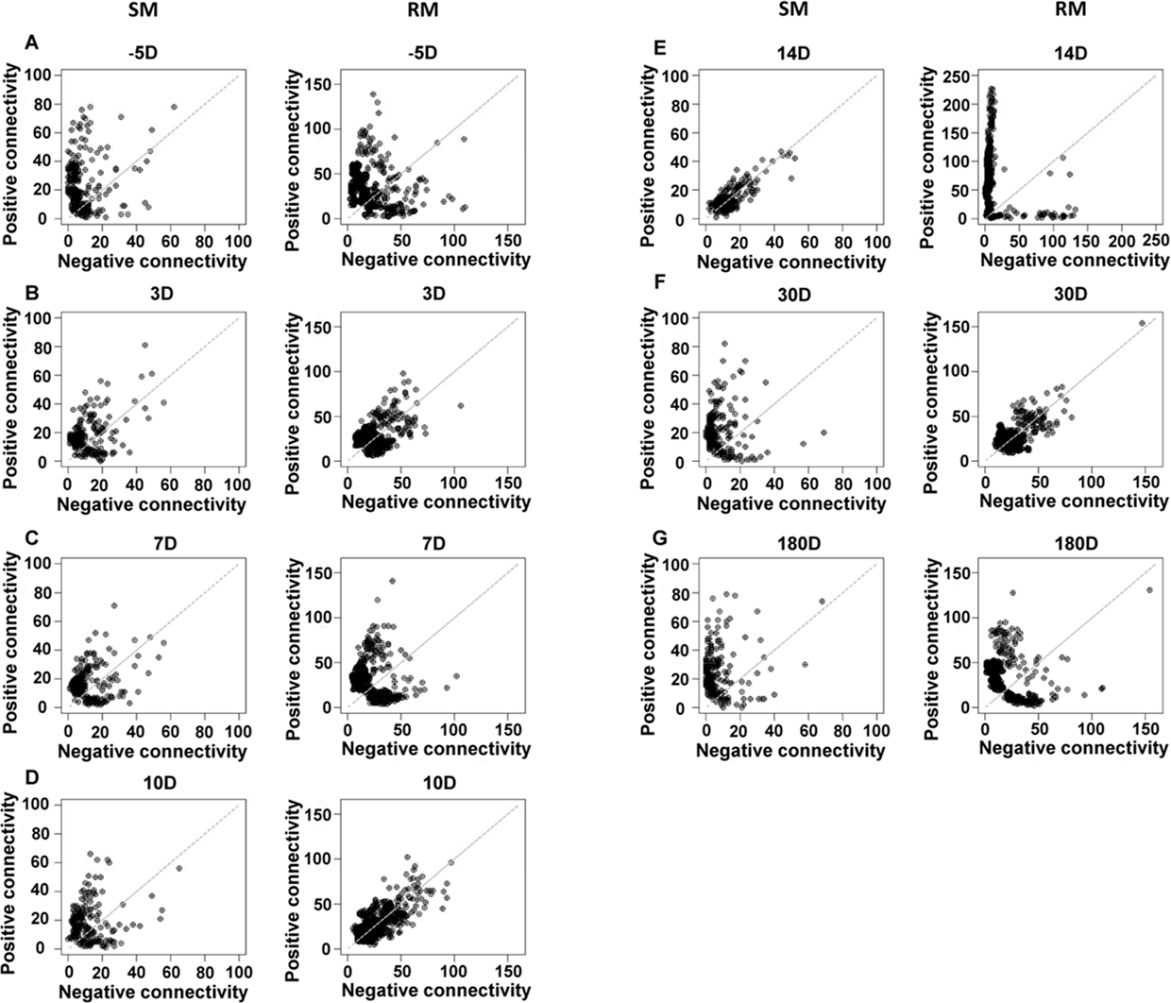
The positive and negative connectivity of genes in SM- and RM- specific GCNs during SIV infection. “-5D”, “3D”, “7D”, “10D”, “14D”, “30D” and “180D” respectively represent 5 days before SIV infection, and 3, 7, 10, 14, 30 and 180 days after SIV infection.

Three SMs and three RMs were selected for the GCN analysis, since they had gene expression data at all the analyzed time points of SIV infection. With the Pearson correlation coefficient (PCC)-based method, fourteen GCNs (7 for SMs and 7 for RMs) were constructed for SMs and RMs at 7 time points of SIV infection (See details in Materials and Methods). The connectivity of a gene in the GCN is composed with two components: positive connectivity and negative connectivity, which respectively represent the numbers of positively and negatively coexpressed genes. The positive and negative connectivity of genes in each GCN are shown in Fig 1. The gray dash line is the diagonal line of the plot, on which genes have the same positive and negative connectivity. At 14 days after SIV infection, SM genes tend to be distributed around the diagonal line, while RM genes tend to be distributed closely to the axis (Fig 1E). While at 10 and 30 days after SIV infection, SM genes tend to be distributed closely to the axis and RM genes tend to be distributed around the diagonal line (Fig 1D and 1F). These results reveal that the coexpression relationships of genes in both SMs and RMs are varied during SIV infection. More importantly, they also indicate that there are extensive differences between the SIV infection of SMs and RMs at the system level from GCNs.

**Fig 2:**
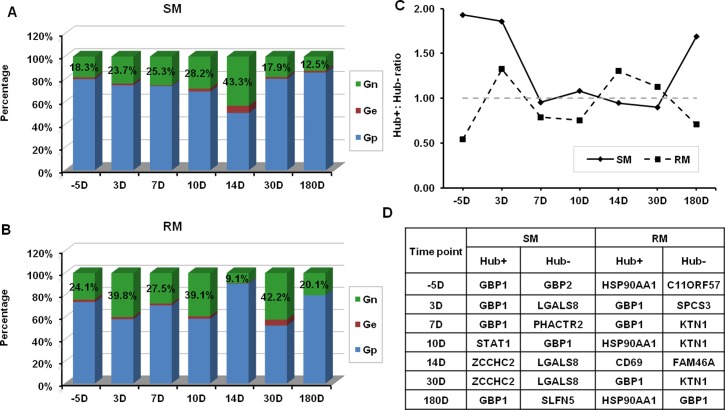
Detailed information of GCNs of SIV infection. (A) Percentages of Gn, Ge and Gp in SM-specific GCNs during SIV infection. (B) Percentages of Gn, Ge and Gp in RM-specific GCNs during SIV infection. (C) The ratios of the number of Hub+ and Hub- genes in SM- and RM-specific GCNs during SIV infection. (D) The Hub+ and Hub- genes with strongest connectivity in SM- and RM-specific GCNs during SIV infection.

The differences are also exhibited with the detailed analysis of genes in SM- and RM-specific GCNs of SIV infection. According to the positive and negative connectivity, genes in the GCN can be grouped into three classes: Gp (positive connectivity > negative connectivity, above diagonal line), Ge (positive connectivity = negative connectivity, on diagonal line) and Gn (negative connectivity > positive connectivity, below diagonal line). The percentages of genes belonging to Gp, Ge and Gn in SM- and RM-specific GCNs are respectively shown in Fig 2A and 2B. In SM-specific GCNs, the percentage of genes belonging to Gn is firstly increased from -5D to 14D, and then decreased from 14D to 180D. While in RM-specific GCNs, the percentage of genes belonging to Gn is increased and decreased more frequently during SIV infection than that in SM-specific GCNs. HSP90AA1, a Hub gene with positive connectivity, is specifically associated with SIV infection in RMs at three time points (-D5, 10D and 180D). There is a Hub gene (LGALS8) with negative connectivity that is only expressed in SIV-infected SMs at three time points (D3, 14D and 30D).

In the groups of Gp and Gn, some genes (i.e., Hub genes) are highly connected. We selected Hub+ and Hub- genes from these two groups with the criteria defined in Materials and Methods, respectively. The ratios of the number of Hub+ and Hub- genes in constructed GNCs are shown in Fig 2C (the hub gene numbers are given in S1 Fig). In SM-specific GCNs, the number of the Hub+ genes is usually larger than that of the Hub- genes. On the contrary, the number of the Hub+ genes is usually smaller than that of the Hub- genes in RM-specific GCNs.

We further investigated genes with highest connectivity in GCNs at different time points of SIV infection (Fig 2D). In RM-specific GCNs, we found 7 unique genes (C11ORF57, CD69, FAM46A, GBP1, HSP90AA1, KTN1, SPCS3). There are also 7 unique genes (GBP1, GBP2, LGALS8, PHACTR2, SLFN5, STAT1, ZCCHC2) found in SM-specific GCNs. GBP1 (guanylate-binding protein-1), which is a major interferon gamma (IFN-γ) induced protein [32], is selected for the gene with highest connectivity in SM- and RM-specific GCNs at several time points of SIV infection. In SMs and RMs, the expression levels of GBP1 are respectively 5.74-fold and 21.56-fold increased at 10 days after SIV infection compared to those before SIV infection (S2A Fig). These results indicate that GBP1 might play an important role of antiviral effect against SIV infection in SMs and RMs. Interestingly, GBP2 is another IFN-γ induced guanylate-binding protein, but it is only up-regulated in SM-specific GCNs. It is found to be an essential immune effector molecule mediating antimicrobial resistance [33]. The gene expression of GBP2 is more than 10-fold increased at the 7 and 10 days after SIV infection when compared with that before infection (S2B Fig). STAT1 is an important transcription factor (TF) that can induce a set of IFN-γ-regulated genes including GBP2 [34]. The fold-change of STAT1 gene expression in SMs is much higher than that in RMs at 10 days after SIV infection (S2C Fig). CD69 is a biomarker of T cell activation. The fold-change analysis of CD69 gene expression (S2D Fig) indicates that the T cell activation is much stronger in RMs than that in SMs at 10 days after SIV infection related to that before infection. Besides these genes, LGALS8, PHACTR2, SLFN5 and ZCCHC2, which are significantly up-regulated in SMs, might be important genes contributing to the BEN phenotypes of SIV/HIV infections [35–37].

### BEN-MAL patterns of expression phenotypes of hub genes in SIV-infected SMs and RMs

**Fig 3:**
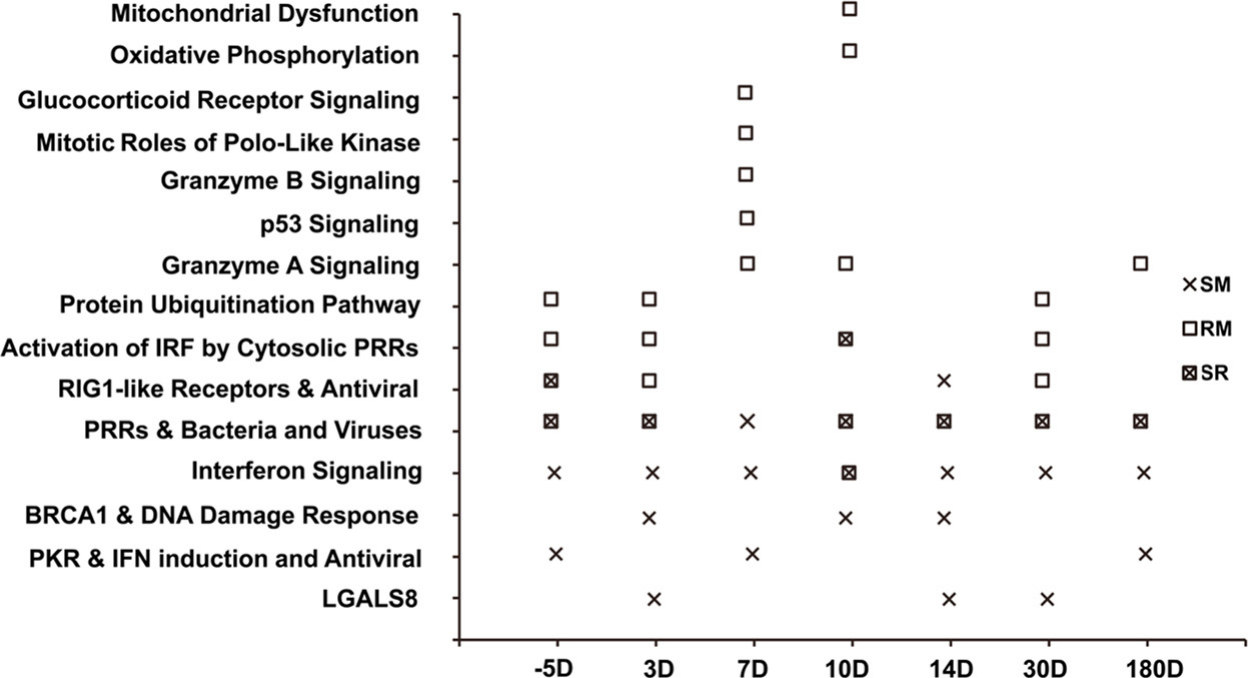
Pathway enrichment analysis of Hub genes in GCNs. “SR” indicates the pathway enriched in both “SM” and “RM”. “PRRs & Bacteria and Viruses” represent the “Role of Pattern Recognition Receptors (PRRs) in Recognition of Bacteria and Viruses”. “PKR & IFN induction and Antiviral” represent the “Role of Protein Kinase R (PKR) in Interferon (IFN) Induction and Antiviral Response” signaling pathway, “BRCA1 & DNA Damage Response” indicate the “Role of the Breast Cancer 1 (BRCA1) gene in DNA Damage Response” signaling pathway.

For Hub genes in each GCN, we further performed the pathway enrichment analysis with the IPA system. Pathways significantly enriched in the Hub genes of GCNs at different time points of SIV infection are shown in Fig 3 (Fisher’s exact test, p-value < 0.01). Several enriched pathways are specifically observed for SMs and RMs. Based on the gene expression phenotypes, these pathways are classified into three different groups: (1) the MAL^+^ phenotypes that are only observed in RMs; (2) the mixed phenotypes (MAL^+^/BEN^+^) present in both RMs and SMs; and (3) the BEN^+^ phenotypes that are solely present in SMs (Fig 3). There are eight enriched pathways in the MAL^+^ group, including the protein ubiquitination pathway, p53, granzyme A signaling, gramzyme B signaling, Mitotic roles of polo-like kinase, Glucocorticoid receptor signaling, oxidative phosyphorylation and mitochondrial dysfunction pathways [38–43]. Another enriched pathway (i.e., granzyme A signaling pathway) is also observed at three time points of SIV infection in RMs. Only two genes (HIST1HIC and PRF1) are involved in this pathway. At the 10 and 14 days of SIV infection, the PRF1 gene expression levels are respectively 4.31-fold and 3.13-fold down-regulated in RMs. These enriched pathways might play an important role in the MAL phenotypes of SIV infection. Specific BEN^**+**^ pattern changes in 4 pathways enriched in Hub genes are identified in SIV-infected SMs. These include the pathways contributing to interferon signaling, BRCA1/DNA damage response, PKR/INF induction and LGALS8 [35,44–46]. These genes were significantly upregulated at two or three time points of SIV infection. There are three enriched pathways (PRR-activated IRF signaling, RIG1-like receptor and PRR pathway) contributing to the mixed (BEN^+^/MAL^+^) phenotypes of SIV infections in RMs and SMs, suggesting that these pathways play a dual role in the host defense against viral infections [44, 47–48].

**Fig 4:**
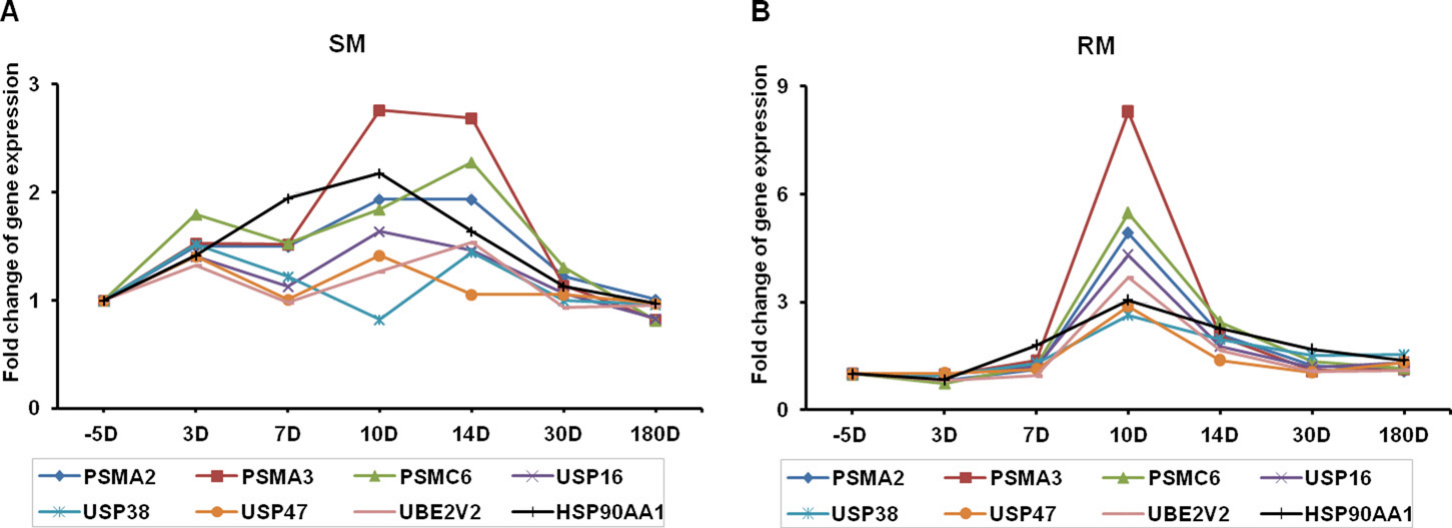
**Fold-changes in gene expression involved in the protein ubiquitination pathway in SMs (A) and RMs (B) after SIV infection (range: 5–180 days).** Eight genes are differentially expressed, including Proteasome subunit alpha (PSMA) type-2 (PSMA2), type-3 (PSMA3), ATPase subunit Rpt4 (PSMC6), ubiquitin specific peptidase 16 (USP16), USP38, USP47, ubiquitin conjugating enzyme E2 variant 2 (UBE2V2) and heat shock protein 90kDa alpha family class A member 1 (HSP90AA1). The p-values in the RM group are statistically significant (p<0.05): PSMA2, p = 0.003; PSMA3, p = 0.003; SMC6, p = 0.03; USP16, p = 0.004; USP38, p = 0.03; USP47, p = 0.002; UBE2V2, p = 0.002; HSP90AA1, p = 0.006.

Of note, the ubiquitin proteasome pathway appeared to be actively involved in SIV infection in RMs at -5, 3 and 30 days. In contrast, they are down-regulated in SMs (1.07-fold and 1.31-fold). Eight genes (PSMA2, PSMA3, PSMA6, USP16, USP38, USP47, UBE2V2, HSP90AA1) contributed to the pattern change in this pathway, which might play a critical role in the degradation of proteins involved in various cellular processes, including inflammatory response, cell proliferation, apoptosis, and so on. The fold-changes in expression of these genes in SMs and RMs during SIV infection are shown in Fig 4. Interestingly, we found that all these genes are rapidly and robustly upregulated at the 10 days after SIV infection in RMs. But in SMs, some of these genes (PSMA2, PSMA3, PSMC6 and HSP90AA1) are slightly upregulated during SIV infection. These findings suggest that proteasomes, the main actors in cellular proteolysis, play important roles in the pathogenesis of HIV/SIV infections.

### Differential expression of immune genes in SIV-infected SMs and RMs

**Fig 5:**
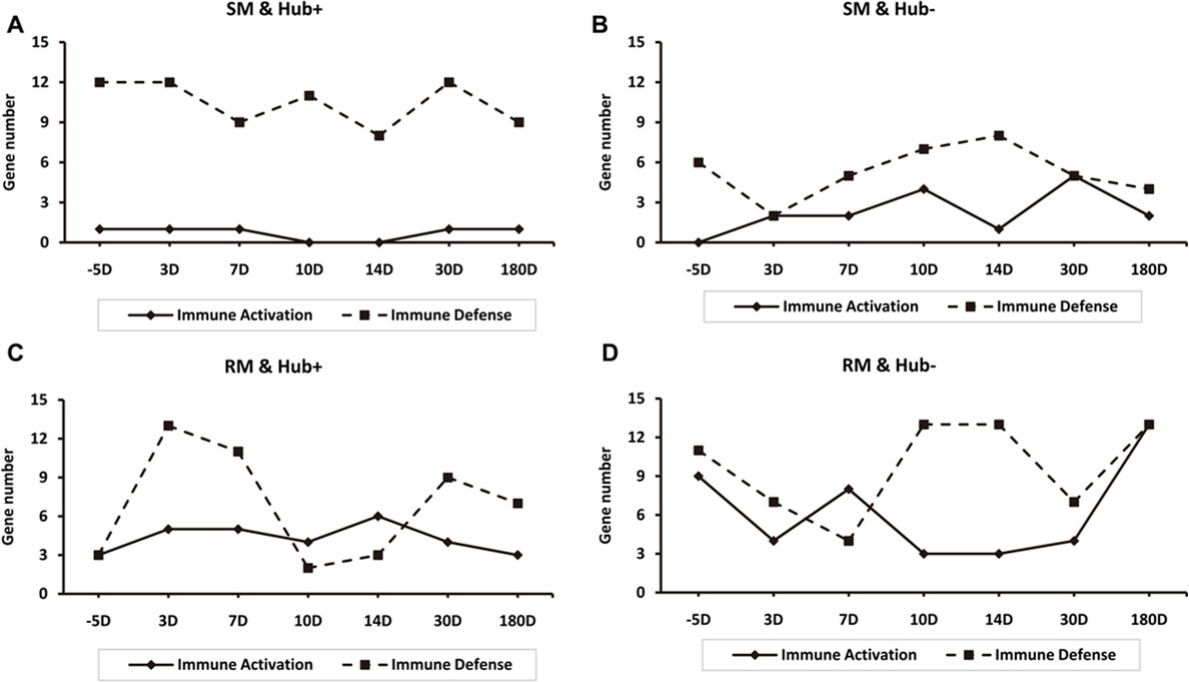
Gene expression patterns of immune activation and immune defense in SMs and RMs after SIV infection (range: 5–180 days). (A) Hub+ genes of SM-specific GCNs. (B) Hub- genes of SM-specific GCNs. (C) Hub+ genes of RM-specific GCNs. (D) Hub- genes of RM-specific GCNs.

Our further investigation was aimed to compare the expression patterns of hub genes that contribute to immune activation and immune defense in SMs and RMs during SIV infection (Fig 5). In the SM-specific GCNs, the number of Hub+ and Hub- immune defense genes is equal to or larger than that of Hub+ and Hub- immune activation genes during SIV infection (Fig 5A and 5B). But there are some exceptions in Hub+ genes of RM-specific GCNs at 10 and 14 days of SIV infection and in Hub- genes of RM-specific GCNs at the 7 days of SIV infection (Fig 5C and 5D). These results concurred with the notion that the limited immune activation in SIV-infected SMs is a mechanism of favoring the preservation of CD4^+^ T-cell homeostasis [8].

### Increased number of transcription factors (TFs) coded by Hub genes in RMs

**Fig 6:**
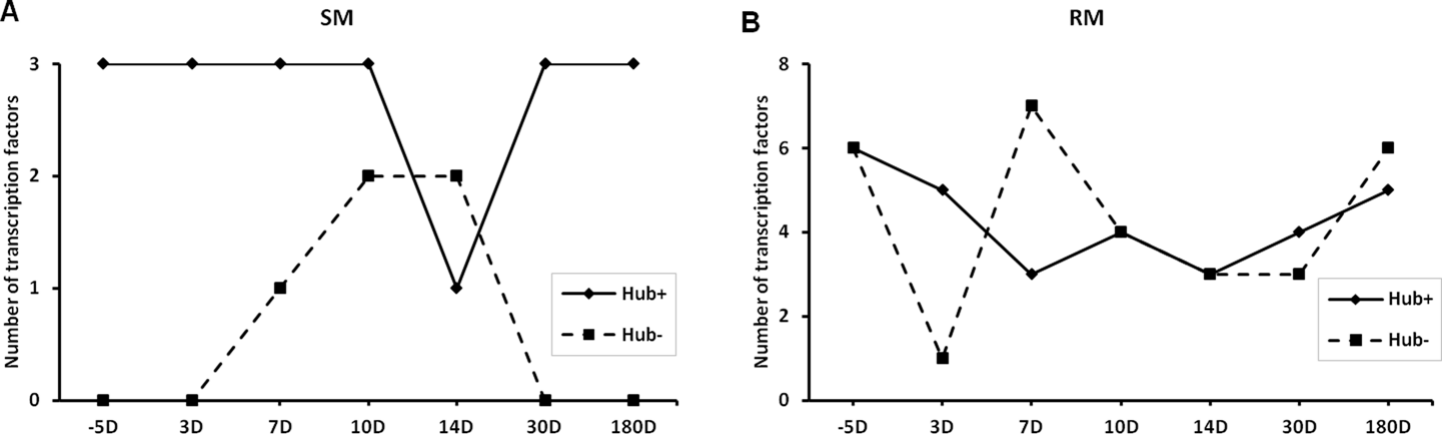
Transcription factors are differentially expressed in SMs (A) and RMs (B) after SIV infection (range: 5–180 days). Both Hub+ and Hub- genes are included.

In the GCN, the Hub+ and Hub- genes are usually subject to different kinds of transcription regulations [49]. We investigate the patterns of TF number included in Hub+ and Hub- genes of SM- and RM-specific GCNs of SIV infection (Fig 6). Three TFs are included in the Hub+ genes of SM-specific GCNs during SIV infection (except 14 days after SIV infection), but TFs are only appeared in Hub- genes of GCNs at the 7, 10 and 14 days of SIV infection. IFI16 and STAT1 are observed at all the analyzed time points of SIV infection, indicating their importance in the SIV infection of SMs. IFI16, which has the ability of detecting the bacterial DNA, also has a role in antiviral innate immune response [50]. STAT1 belongs to the Hub- gene at the 14 days of SIV infection, but belongs to the Hub+ gene at other time points of SIV infection. This implicates that the coexpression relationship between STAT1 and other genes might be largely changed. STAT1 is also an important TF in the SIV infection of RM. Different from the SMs, STAT1 is a Hub- gene in the RM-specific GCN at the 10 days after SIV infection, and a non-Hub gene in the RM-specific GCN at the 14 days after SIV infection. SMAD5 belongs to the Hub+ gene of GCNs at all considered time points of SIV infection. SMAD5 (SMAD family member 5) is associated with the transforming growth factor β (TGF- β) anti-proliferative effects [51].

### Correlation between Hub genes and changes in CD4^+^T cells in SIV-infected RMs and SMs

**Fig 7:**
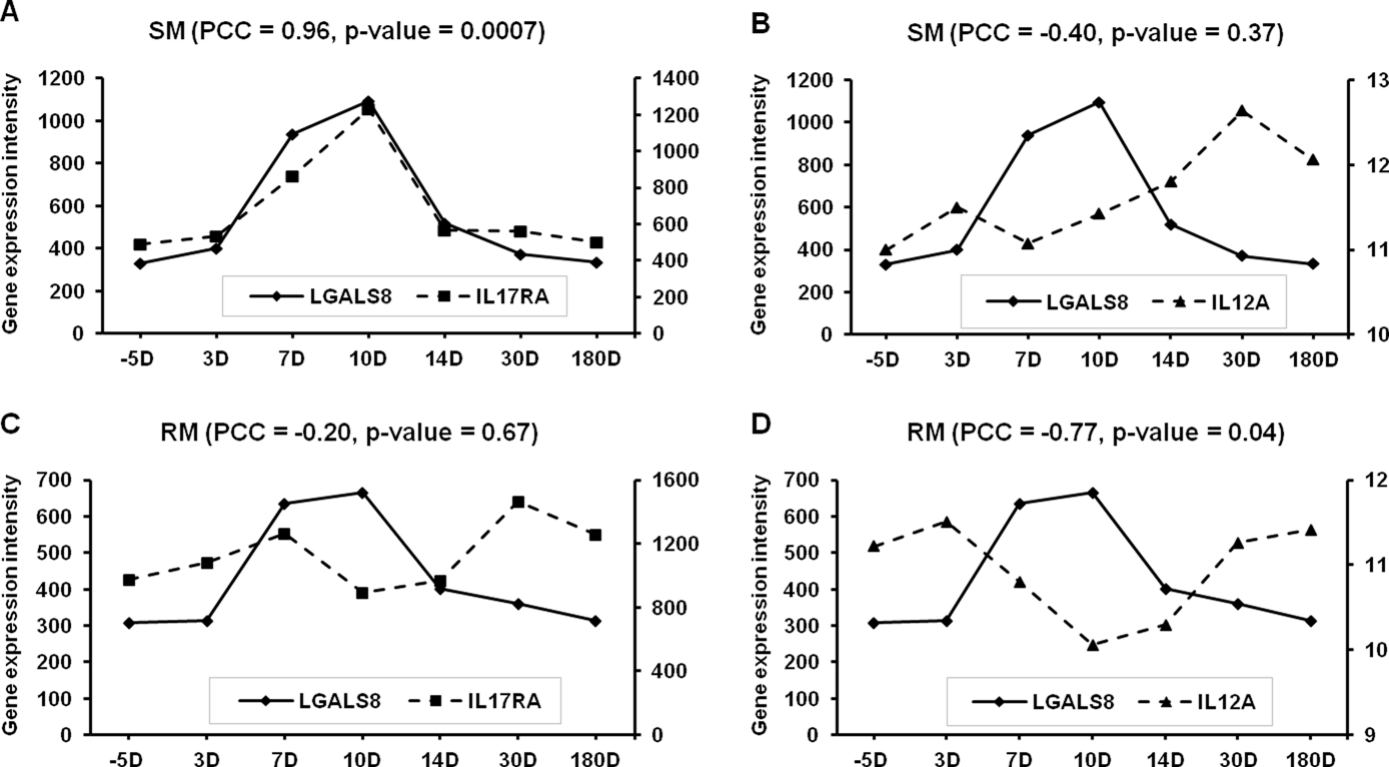
**Pearson correlation between the gene expression levels of LGALS8, IL17RA and IL12A in SMs (A & B) and RMs (C & D) after SIV infection (range: 5–180 days).** A & C: Galectin-8 (LGALS8) and interleukin 17 receptor A (IL17RA); B & D: LGALS8 and interleukin 12A (IL12A).

**Fig 8:**
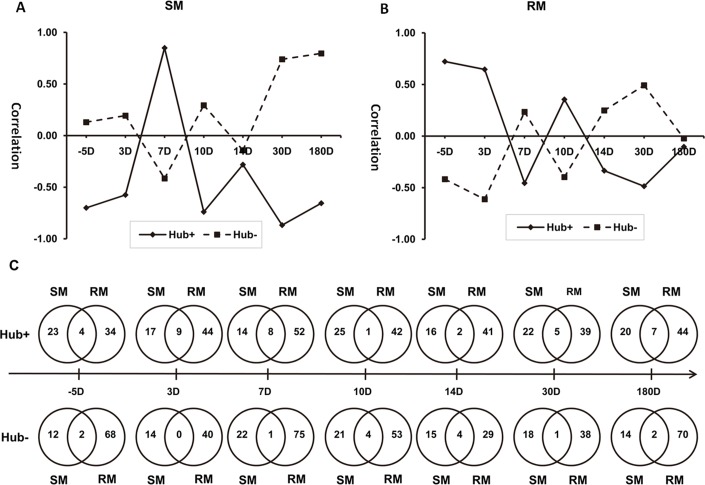
Pearson correlation between Hub+ and Hub- gene expression patterns. (A) Hub genes in SM-specific GCNs. (B) Hub genes in RM-specific GCNs. (C) Overlapping gene expression patterns of Hub+ and Hub- genes in SMs and RMs after SIV infection (range: 5–180 days). The frequency of the overlapping Hub+ genes (1–9) is significantly higher than that of the Hub- genes (0–4).

Connectivity analysis of the SM- and RM-specific GCNs reveals that there are extensive differences between the SIV infection of SMs and RMs. For the highly connected genes (i.e., Hub genes), pathway enrichment analysis shows that SMs exhibit a strong innate immune response to SIV viral infections. We further examined if expression patterns of Hub genes are correlated with changes in phenotypes and numbers of CD4+T cells in SIV-infected RMs and SMs. Detailed analysis of Hub+ and Hub- genes indicates that differential transcription regulations of LGALS8, IL17RA and IL12A exist in SMs and RMs during SIV infection (Fig 7 and S3 and S4 Figs). The immune functions of these genes are associated with the phenotypes of CD4+ T cells [52–55], suggesting that LGALS8, IL17RA and IL12A may contribute to the pathogenesis and therapeutics of HIV/SIV infection. Further analysis of the Pearson correlations between Hub+ and Hub- genes also reveals the differences in gene expression patterns in SIV-infected SMs and RMs (Fig 8A–8C). Further investigation of these differences might help to understand the pathogenesis of SIV/HIV infection.

## Discussion

A number of comparative studies have reported the extreme phenotypes of SIV infection in RMs and SMs suggesting that in addition to causing abnormalities in the adaptive immune response, the viral infection also has a significant impact on essential components of the innate immune system that may further contribute to the remarkable phenotypic changes [7–10, 24–27]. In this study, we first reported the meta-analysis of double connectivity of GCNs toward the study of molecular mechanisms of SIV/HIV diseases by using the gene expression data generated from the SIV-infected SMs and RMs [11,23]. Comparative double connectivity analysis of SM- and RM-specific GCNs at different time points of SIV infection reveals some novel insights that have not previously been reported.

For genes with the highest connectivity in the Groups of Gn and Gp, several genes (LGALS8, PHACTR2, SLFN5, STAT1, ZCCHC2) are only observed in SM-specific GCNs. Further analysis of these genes might gain more insights of the SIV infection. It has been shown that these genes may contribute to the host defense against various diseases including microbial infections and cancer [35–37]. For example, LGALS8 (galectin 8), which has recently been found as a bactericidal lectin, is involved in the host innate immunity [35]. These genes might have an important effect on the BEN phenotypes of SIV/HIV infections. According to the molecular interaction information in the Ingenuity Pathway Analysis (IPA) knowledge base [56], we found the LGALS8 might be associated with the IL-17 receptor (IL17RA) and IL-12 (IL12A). IL-17 is produced by the Th17 cell. IL-12 is a cytokine which is crucial for differentiating naive CD4+ T cell to the Th1 pathway [55]. The gene expression of LGALS8 is significantly positive correlated with that of IL17RA in SMs, but the gene expression of LGALS8 is significantly negatively correlated with that of IL12A in RMs. LGALS8 might play a role in altering the balance between Th17 and Th1 cells in the infection of SIV disease [53].

We investigated in detail three groups of genes associated with three phenotypes (BEN^+^, MAL^+^ and mixed BEN^+^/MAL^+^) identified through a comparative double connectivity analysis of the natural SM and non-natural RM host transcriptomic data. Assessment of enrichment of gene ontology terms for gene expression patterns with nominally significant p values for each of the three phenotypes showed enrichment in several signaling pathways. Four signaling pathways (interferon signaling, BRCA1/DNA damage response, PKR/INF induction and LGALS8), which may control the viral infection, are clearly associated with the BEN^+^ phenotype in SMs. Among the members of the interferon regulatory factor (IRF) family, IRF1 is one of the central mediators of both the innate and adaptive immune responses, which are required for antigen processing/presentation, Th1/Th2 differentiation, and the immune activities of natural killer (NK) cells and macrophages [57]. PKR is a key component of the IFN-associated innate antiviral defense pathway in mammalian cells [46]. BRCA1, a critical regulator of DNA damage repair and cell survival [45], might attenuate the sequelae of SIV/HIV infections. LGALS8, a danger receptor that restricts bacterial proliferation [35], may have antiviral activity against HIV/SIV infections. During early SIV/HIV infection, IRF1 and related signaling pathways (RIG1-like receptor- and PRR-mediated pathways) may play a dual role that is critical for driving viral replication as well as eliciting antiviral responses [44, 47–48]. Therefore, these factors can contribute to the mixed (BEN^+^/MAL^+^) phenotypes of SIV infections through differential regulatory mechanisms. Among the eight enriched genes or pathways in the MAL^+^ group [the ubiquitin proteasome system (UPS), p53, granzyme A signaling, gramzyme B signaling, Mitotic roles of polo-like kinase, glucocorticoid receptor signaling, oxidative phosyphorylation and mitochondrial dysfunction], mitochondrial signaling may be the central player that contributes to the pathogenesis of HIV/SIV infections with coordination of the other seven signaling partners [38–43].

Mitochondria is an intracellular organelle that arose from bacteria entering a eukaryotic cell to form a symbiotic relationship. This organelle is now recognized not only as the main intracellular source of energy by converting the potential energy of food molecules into ATP, but also as a major controller in many cellular pathways, including the pathways contributing to the MAL^+^ phenotype of SIV infection, autophagy/mitophagy and apoptosis, which regulate the turnover of organelles and proteins within cells, and of cells within organisms, respectively [58]. Proteins from HIV have been implicated in acting on mitochondria to delete the targeted key immune cells, which results in viral evasion of the immune system and progresses to AIDS [59]. It has been suggested that the exo- (e.g, microbiota) and endo- (e.g., mitochondria) symbiotic balance (EESB) is essential for health and that the exo-endo Sym imbalance plays an important role in the pathogenesis of infectious diseases, including HIV/AIDS [17–18]. There are two significant factors, microbial translocation across the gut barrier (disturbing exosymbiosis) and mitochondria mediated apoptosis (dysregulation of endosymbiosis), which have contributed to the pathogenesis of AIDS caused by HIV and SIV [19–21]. The GCN analyses in this study associate the MAL^+^ phenotype with an enrichment for linked to dysfunctions of mitochondrial signaling and related pathways. The genes LGALS8 and IL-17RA, which positively regulate the barrier function of the gut mucosa, are significantly down-regulated in RMs when compared to SMs within 7 to 10 days after SIV infection. Our findings concurred with previous studies in that SIV-induced IL-17 deficiency could promote bacterial translocation from the gut and LGALS8 could suppress the viability of targeted bacteria [60–61]. It has also been shown that lower levels of mucosal T cells secreting IL-17 are associated with AIDS progression, dysbiosis of the gut microbiota, increased apoptosis and higher levels of T cell activation in HIV-or SIV-infected subjects [62–63]. As progression to AIDS is dynamic, the time-series experiments with SIV-infected SMs and RMs enable the identification of Hub genes contributing to the pathogenesis of AIDS. HSP90AA1, a Hub gene with positive connectivity, is only expressed in SIV-infected RMs at multiple time points. SP90AA1 has been recently detected as a hub node in patients with HIV-associated encephalitis [64]. Another Hub gene (LGALS8) with negative connectivity is specifically associated with SMs at the early stage (3, 14 and 30 days) of SIV infection. LGALS8 could modulate the neutrophil function related to transendothelial migration and microbial killing [65]. It remains unclear whether these two Hub genes contribute to modulation of EESB. Taken together, these results suggest that there is a mechanistic link between dysbiosis of the gut microbiota (exosymbiotic disorder) and mitochondrial dysfunction (endosymbiotic abnormalities) in HIV/SIV infections. These findings support the notion that the exo-endo Sym imbalance (EESI) may play an important role in pathogenesis and management of infectious diseases, including AIDS caused by HIV-1 and SIV. These findings also emphasize the interchange between the organism and its ecological environments with more holistic consideration of immune regulation. Dissecting the mechanisms of the EESB that maintains immune homeostasis and the EESI problems in HIV/SIV infections may lead to a better understanding of the pathogenesis of AIDS and the development of novel interventions for the rational control of this disease.

## Materials and Methods

### Ethics statement

The animal study was performed in strict accordance with the recommendations in the Guide for the Care and Use of Laboratory Animals of the National Institutes of Health. Our protocols were approved by the Institutional Animal Care and Use Committee (IACUC) of Emory University and the University of Pennsylvania (permit No. A3180-01). All surgery was performed under anesthesia with ketamine or Telazol, and all efforts were made to minimize suffering [11]. There were no unexpected deaths in this study [11].

### Animals and SIV infection

A total of 18 nonhuman primates were included in this study, of which 17 underwent full analysis. Five SMs and five RMs were inoculated with an uncloned SIVsmm derived from an experimentally infected SM at 11 days after infection (1 ml of plasma) as described previously [11]. One RM was excluded from the analysis due to absence of robust virus replication. In addition, eight RMs were inoculated i.v. with SIVmac239. In the SIV-infected animals, the average durations of infection were 12.0 ± 0.5 years for SMs, as estimated by the date of the first SIV-positive test, and 0.8 ± 0.1 years for RMs. The sooty manbabeys and rhesus macaques in this study were infected with SIVsmm in two separate mixed-species cohort in Oct and Dec 2005. The current study focused on SIV infections during the first 0.5 year. Blood collection was performed by venipuncture. For lymph node (LN) biopsy, animals were anesthetized with Ketamine or Telazol. LN biopsies were taken from 2 SIVsmm-infected SMs and 2 SIVmac239-infected RMs for each interval. All animals used in this study were housed at the Yerkes National Primate Research Center in accordance with the regulations of the merican Association of Accreditation of Laboratory Animal Care standards. All animals were in single cage housing during the study as per IACUC approval. The feeding regimens for all nonhuman primates in this study was provision of chow twice daily as well as additional enrichment foods at certain time intervals, and is in compliance with the Animal Welfare Act. All animals in this study were enrolled in a Nonhuman Primate Enrichment Program for that is locally IACUC approved and in compliance with the Animal Welfare Act. The feeding regimens for all nonhuman primates also is covered by a Center SOP (SOP 4.8) describing provision of chow twice daily as well as additional enrichment foods at certain time intervals. At the conclusion of this study, mangabeys were re-assigned to other projects per the Yerkes Research Animal Access Committee (RAAC). At the conclusion of this study, rhesus macaques were re-assigned to other AIDS studies at Yerkes and received continual veterinary monitoring, and eventual euthanasia by pentabarbitol (100mg/kg) injection. Rhesus were anesthetized with ketamine 10mg/kg or Telazol 4–5 mg/kg prior to euthanasia.

### Microarray Dataset

We analyzed the transcriptome-wide gene expression data from several SMs and RMs infected with the same SIV strain (i.e., SIVsmm) [11]. The gene-expression profiles of monkeys at seven time points of SIV infection (i.e., 5 days before infection, and 3, 7, 10, 14, 30 and 180 days after infection) were measured with the Affymetrix GeneChip Rhesus Macaque Genome Arrays, which contained more than 47,000 transcripts.

The microarray dataset analyzed in this study was generated in experimental infections of five SMs and four RMs with the same SIV viral strain (i.e., SIVsmm) [11]. The RNA samples derived from whole blood were collected at different time points of SIVsmm infection (5 days before infection, and 3, 7, 10, 14, 30 and 180 days after infection). The transcriptome data in these samples were measured with the Affymetrix GeneChip Rhesus Macaque Genome Arrays, which contains over 47,000 transcripts. The expression values can be obtained from the NCBI GEO database (http://www.ncbi.nlm.nih.gov/geo/, accession number GSE16147). The CD4+ T cell counts on whole blood were also measured at these time points using the flow cytometry. A detailed description of the experiments can be found in the original paper [11].

### Identification of differentially expressed genes and function annotation

Using the robust multichip average (RMA) normalized microarray data, differentially expressed genes were determined based on the fold change method and the p-value of one-way analysis of variance (ANOVA) model adjusted by the Benjamini-Hochberg multiple testing correction [66]. With the criteria of fold change ≥2 and the p-value ≤ 0.0008342, Bosinger and colleagues have picked up 428 probes with differential expression during SIV infection in SMs [11]. In this study, we further identified 941 differentially expressed probes in RMs with the same approaches (the fold change ≥2; the p-value ≤ 0.05) using Partek Genomics Suite software v6.5 (Partek Inc). These selected probes were mapped to gene symbols using the DAVID bioinformatics resources [67] and the Ingenuity Pathway Analysis (IPA) system [56]. The functional categories of these genes were further annotated with the DAVID bioinformatics resources, the IPA software and literature examination. The probes without gene symbol annotation were not included in the following analysis.

### Determination of miRNA target genes and transcription factors

The annotation information from miRTarbase database (v2.4, released on 04/15/2011) [68] and miRNA target predictions from TargetScan website (v5.1, http://www.targetscan.org/) [69] were used to determine whether the analyzed genes were miRNA targeting or not. The miRTarbase is a high-quality database which collects about 4,000 experimentally validated miRNA-target interactions. For TargetScan, only the reliable predictions (i.e., conserved gene transcripts targeting with conserved miRNAs, and context score percentile > 50) were considered.

The transcription factors (TFs) were obtained from the annotation information from IPA system, Entrez gene database (http://www.ncbi.nlm.nih.gov/gene/), and the TF collection in previous literature [70].

### Pathway enrichment analysis

To further define the mechanistic connection between the BEN and MAL phenotypes of SIV infections we performed the pathway enrichment analysis using the gene expression data generated from the SIV-infected SMs and RMs [11]. The experiment was designed to compare gene expression in SIV-infected SMs and RMs. We performed the pathway enrichment analysis with the IPA system. With the Fisher’s exact test method, the IPA system identified pathways which were statistically significant to a set of genes. The significance level (i.e., the p-value of Fisher’s exact test) indicates the likelihood that the pathway would be indentified by random chance. In this study, the significant pathways were defined as those with p-value ≤ 0.01 and the number of focus genes ≥ 2.

### Network reconstruction

The gene coexpression networks (GCNs) were respectively reconstructed for SM and RM at different time points of SIV infection. In a GCN, the nodes represent genes, and the edges connect two coexpressed genes. For a given time point of SIV infection in SM, the Pearson correlation coefficient method was firstly used to measure the similarity of gene expression profiles between any pair of probes. The PCC value between probes x and y can be computed with the following equation:

$$r=\frac{\sum_{i=1}^{n}\left(x_{i}-\bar{x})(y_{i}-\bar{y}\right)}{\sqrt{\sum_{i=1}^{n}{\left(x_{i}-\bar{x}\right)}^{2}}•\sqrt{\sum_{i=1}^{n}{\left(y_{i}-\bar{y}\right)}^{2}}}$$

where n (n≥ 3) is the total number of samples at a given time points, *x*_*i*_ and *y*_*i*_ are expression levels of x and y in the ith sample, respectively, and $\bar{x}$ and $\bar{y}$ are means of expression levels among samples, respectively. The PCC value can be ranged from -1.0 (completely negative correlation) to 1.0 (completely positive correlation). The statistical significance of PCC value was then assessed using the result that the statistic $t=r\sqrt{(n-2)/(1-r^{2}})$ has a Student’s t-distribution with *df* = *n*-2 under the null hypothesis of no correlation [49,71]. Finally, we connected genes with significantly PCC values in the GCNs, and defined the weight of each edge with the PCC value of connected two genes. For genes with two more probes, the PCC value indicating the highest connection was chose for the edge weight.

For a given GCN, the connectivity of a gene is usually defined as the total number of its corresponding edges, and is consisted with two components: positive and negative connectivity, while considering the algebraic sign of PCC value [49]. Following the method presented in [49], we defined one gene i as a Hub- gene if it satisfied the following criteria: (1) *X*_(i-)_ > *X*_(i+)_; (2) *X*_(i-)_ > T-, where *X*_(i-)_ and *X*_(i+)_ respectively represent the negative and positive connectivity of the analyzed gene, T- is the threshold value and can be computed with the equation: T- = <*X*-> + 1.4SD_x-_, <*X*-> and SD_x-_ are the average and standard deviation of negative connectivity in the analyzed GCN, respectively. Similarly, the Hub+ genes can be defined with the criteria: (1) *X*_(i+)_ > *X*_(i-)_; (2) *X*_(i+)_ > T+. Since the GCNs were constructed with the significantly expressed genes, a slightly low factor 1.4 was set in this study for the selection of Hub genes. According to this method, Hub genes (including the Hub- and Hub+ genes) can be obtained for each GCN.

In this study, the molecular interaction networks were also constructed for LGALS8 and its coexpressed genes in SM- and RM-specific GCNs with the IPA system. The IPA Knowledge Base is a high-quality database collecting the interaction information published in previous publications. Based on the interaction information, the IPA system algorithmically assembles the molecular interaction networks with the input genes and other molecules contained in the IPA Knowledge Base. The IPA system also scores each network based on the number of genes included in the input gene set. The network score is the negative log of Fisher's exact test P value, which measures the probability of the focus genes in a given network by random chance [72].

### Database

The Gene IDs in Entrez gene database are listed as follows: C11ORF57, *Macaca mulatta*, 711168; CD69, *Macaca mulatta*, 717288; FAM46A, *Macaca mulatta*, 693306; GBP1, *Macaca mulatta*, 694538; HSP90AA1, *Macaca mulatta*, 708431; KTN1, *Macaca mulatta*, 697744; SPCS3, *Macaca mulatta*, 100429721; PSMA2, *Macaca mulatta*, 701683; PSMA3, *Macaca mulatta*, 700251; PSMC6, *Macaca mulatta*, 710822; USP16, *Macaca mulatta*, 706403; USP38, *Macaca mulatta*, 700235; USP47, *Macaca mulatta*, 701660; UBE2V2, *Macaca mulatta*, 714735; HSP90AA1, *Macaca mulatta*, 708431; LGALS8, *Macaca mulatta*, 710375; IL17RA, *Macaca mulatta*, 709005; IL12A, *Macaca mulatta*, 703205; GBP1, *Cercocebus atys*, 105593619; GBP2, *Cercocebus atys*, 105593623; LGALS8, *Cercocebus atys*, 105574976; PHACTR2, *Cercocebus atys*, 105600340; SLFN5, *Cercocebus atys*, 105589355; STAT1, *Cercocebus atys*, 105579958; ZCCHC2, *Cercocebus atys*, 105590002; PSMA2, *Cercocebus atys*, 105596930; PSMA3, *Cercocebus atys*, 105586681; PSMC6, *Cercocebus atys*, 105586626; USP16, *Cercocebus atys*, 105571857; USP38, *Cercocebus atys*, 105580776; USP47, *Cercocebus atys*, 105595701; UBE2V2, *Cercocebus atys*, 105592463; HSP90AA1, *Cercocebus atys*, 105596717; IL17RA, *Cercocebus atys*, 105591278; IL12A, *Cercocebus atys*, 105598171.

## Supporting Information

S1 Fig Number of Hub genes in SM- and RM-specific GCNs of SIV infection at different time points. (TIF)

S2 Fig Fold change of averaged gene expression of GBP1 (A), GBP2 (B), STAT1 (C) and CD69 (D) during SIV infection in SMs and RMs.
(TIF)

S3 Fig LGALS8 related molecular interaction network in SMs. There is an interaction LGALS8 and IL17R, which positively regulate the barrier function of the gut mucosa. LGALS8 may contribute to the regulation of PDE6H and PDE8B.
(TIF)

S4 Fig A LGALS8 and IL12R-related network module in SIV-infected RMs. The gene expression of LGALS8 is significantly negatively correlated with that of IL12A in RMs.
(TIF)
