# Supplementary figures and images for: Coexpression Network Analysis of Benign and Malignant Phenotypes of SIV-Infected Sooty Mangabey and Rhesus Macaque
Source: PLoS One. 2016 Jun 9;11(6):e0156170. doi: 10.1371/journal.pone.0156170 (PMC4900581; doi:10.1371/journal.pone.0156170)

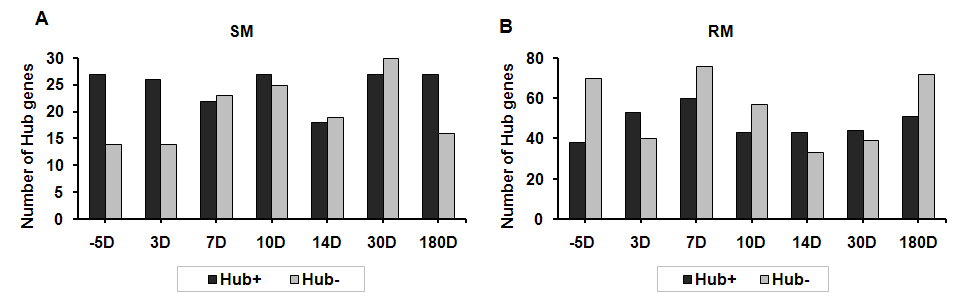

Supplement: S1 Fig — (TIF) [file pone.0156170.s001.tif]

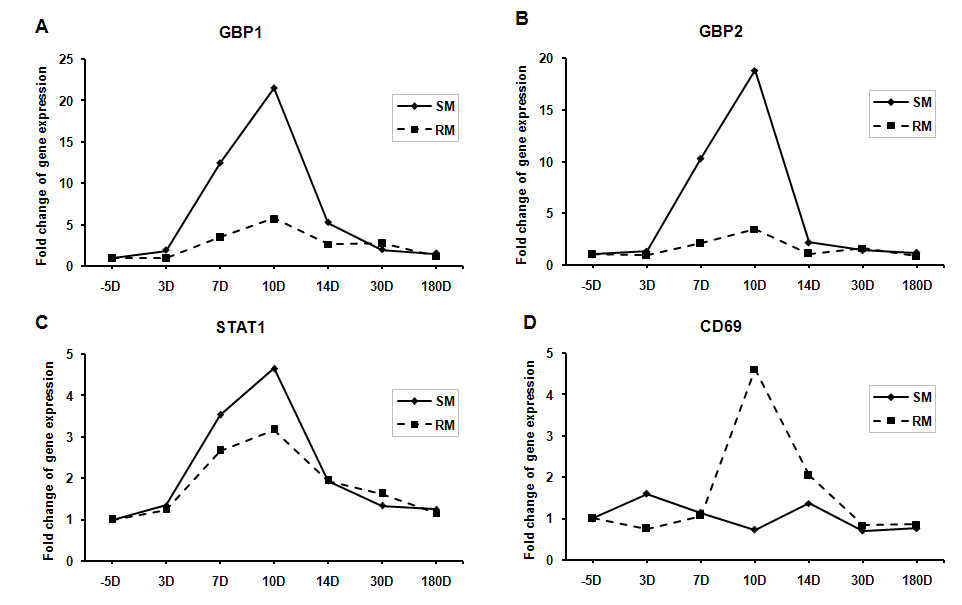

Supplement: S2 Fig — Fold change of averaged gene expression of GBP1 (A), GBP2 (B), STAT1 (C) and CD69 (D) during SIV infection in SMs and RMs. (TIF) [file pone.0156170.s002.tif]

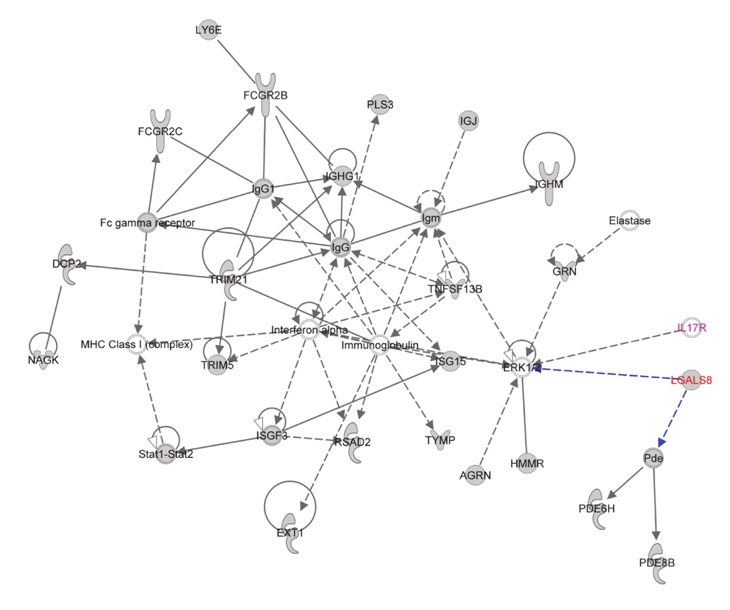

Supplement: S3 Fig — There is an interaction LGALS8 and IL17R, which positively regulate the barrier function of the gut mucosa. LGALS8 may contribute to the regulation of PDE6H and PDE8B. (TIF) [file pone.0156170.s003.tif]

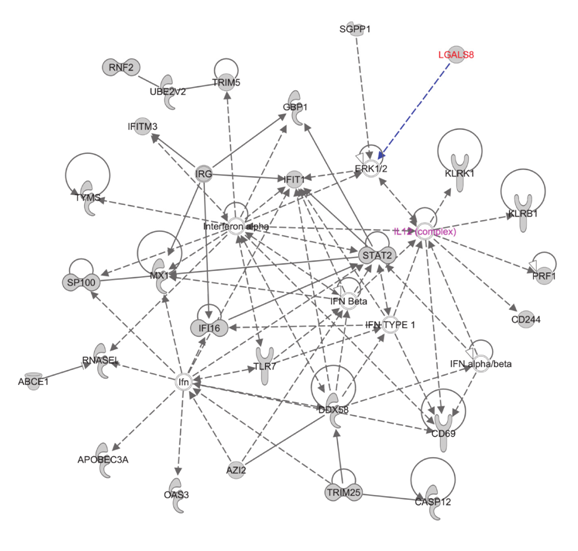

Supplement: S4 Fig — The gene expression of LGALS8 is significantly negatively correlated with that of IL12A in RMs. (TIF) [file pone.0156170.s004.tif]
